# Tailorable Electronic and Electric Properties of Graphene with Selective Decoration of Silver Nanoparticles by Laser-Assisted Photoreduction

**DOI:** 10.3390/nano12203549

**Published:** 2022-10-11

**Authors:** Inseon Song, Yujeong Kim, Byung Hoon Lee, Minji Chae, Sooyeon Kim, ChangKyu Yoon, Min-Kyu Joo, Jeeyoung Shin, Soo Min Kim, Changhyun Ko

**Affiliations:** 1 Department of Applied Physics, College of Engineering, Sookmyung Women’s University, Seoul 04310, Korea; 2 Center for Integrated Nanostructure Physics (CINAP), Institute for Basic Science (IBS), Sungkyunkwan University, Suwon 16419, Korea; 3 Department of Mechanical Systems Engineering, Sookmyung Women’s University, Seoul 04310, Korea; 4 Institute of Advanced Materials and Systems, Sookmyung Women’s University, Seoul 04310, Korea; 5 Department of Chemistry, Sookmyung Women’s University, Seoul 04310, Korea

**Keywords:** graphene, two-dimensional material, silver nanoparticle, photoreduction, field-effect transistor, doping effects

## Abstract

While graphene shows great potential for diverse device applications, to broaden the scope of graphene-based device applications further, it would be necessary to tune the electronic state of graphene and its resultant electrical properties properly. Surface decoration with metal nanoparticles is one of the efficient doping methods to control the properties of two-dimensional materials. Here, we report the p-type doping effects in single-layer graphene decorated with silver nanoparticles (AgNPs) that were formed area-selectively by the facile one-step photoreduction (PR) process based on focused-laser irradiation. During the PR process, AgNPs were reduced on graphene in AgNO_3_ solution by laser-driven photoexcitation followed by chemical reactions. Based on scanning electron microscopy analyses, the morphology characteristics of AgNPs were shown to be modulated by the laser dwell time and power controllably. Further, p-type doping effects were demonstrated using graphene-field-effect transistor structures whose graphene channels were selectively decorated with AgNPs by the PR process, as validated by the decrease in channel resistance and the shift of the Dirac point voltage. Moreover, the growth of AgNPs was observed to be more active on the graphene channel that was laser-annealed ahead of the PR process, leading to enhancing the efficiency of this approach for altering device characteristics.

## 1. Introduction

Graphene, a monatomic sheet of a honeycomb crystal structure composed of sp^2^-hybridized carbon atoms, has been studied extensively for a couple of decades since it does not only show fundamentally unique phenomena including unordinary valley structures and quantum Hall effects [1] but it also has many beneficial physical properties such as ultrahigh charge carrier mobility [2], superb mechanical strength and elastic modulus [3,4], high transparency [5], etc., leading to a variety of potential applications such as electronic and optoelectronic devices [6,7,8,9], electrical energy storage [10], flexible electronics [11], metastructures [12], and so forth. Particularly for electronic and optoelectronic graphene-based devices, to broaden the scope of their applications further, it is highly required to tune the carrier concentration and majority carrier type of graphene, as the doping level of conventional semiconductors should be tailored in a wide range to meet the differing needs of contemporary electronics and optoelectronics [13].

From this perspective, although it is quite challenging to control the electronic properties in two-dimensional (2D) structures, various approaches have been developed for doping 2D materials such as substitutional doping [14,15], electrostatic gating [8], surface functionalization [16,17], gas adsorption [13], ion irradiation [18], intercalation [19,20], and so on. The growth of metal nanoparticles on the graphene surface is also one of the effective doping methods to modulate the electronic state of graphene properly via charge carrier transfer across metal/graphene interfaces [21]. Among various metal nanoparticles, due to the extraordinary physical and chemical properties of silver nanoparticles (AgNPs), the 0D/2D heterostructures of AgNPs/graphene have been employed for demonstrating diverse synergetic applications such as electrocatalysts [22,23,24], electrochemical energy storages and sensors [25], photodetectors [26], transparent conductive coatings [27,28], etc., as well as for exploring fundamental phenomena such as localized surface plasmon resonance [21,26,29]. Moreover, integrating AgNPs with graphene structures may be structurally suitable for flexible device applications [30,31,32]. Meanwhile, a few studies have reported that AgNP decoration induces n-type doping in graphene [21,26,29,33].

While AgNPs have been produced on graphene in various ways such as chemical reduction, gas phase synthesis, thin film deposition followed by heat treatment, etc. [21,22,25,26,29,34], to our knowledge, no previous study has been reported regarding AgNP decoration on graphene by the photoreduction (PR) process driven by laser irradiation. This method enables the formation of AgNPs on 2D materials controllably via modulating the laser processing conditions [35]. Recently, Y. Lee, et al. have demonstrated controllable p-type doping in 2D molybdenum disulfide (MoS_2_), one of the highly attractive 2D semiconductor species, by AgNP decoration based on the laser-assisted PR method [35]. Since graphene is a gapless semi-metal, AgNP growth can be activated by direct photoexcitation more easily on graphene than on a 2D semiconductor. Further, by adopting a focused-laser beam as an irradiation source for the PR process, AgNPs can be selectively formed on a desired area of graphene [13,36]. This one-step process may even allow microscale complicated patterns of AgNPs to be written on graphene without any additional lithography and subsequent high-temperature annealing processes required for the conventional area-selective fabrication of AgNPs based on physical deposition techniques [13,26,35,36,37,38].

In this work, utilizing the facile one-step focused-laser-assisted PR process, p-type doping effects were achieved in graphene where AgNPs were synthesized area-selectively on its surface. It was shown that the morphology characteristics of AgNPs on graphene were adjustable by tuning the laser processing parameters. Adopting graphene-based field-effect-transistor (GFET) devices, we demonstrated that the electronic state and electrical characteristics of graphene could be modified properly by AgNP-decoration-driven p-type doping. In addition, it was also observed that focused-laser-assisted annealing on graphene in water, prior to the PR process, made the AgNP growth kinetics more active, resulting in a more significant alteration of the device characteristics.

## 2. Materials and Methods

### 2.1. Nanoparticle Synthesis and Characterization

**Figure 1:**
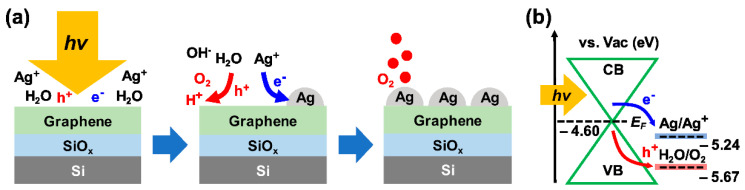
(**a**) Schematic drawing and (**b**) energy band diagram of the photoreduction (PR) process of silver nanoparticles (AgNPs) on graphene in the AgNO_3_ solution. The electron energy levels are indicated with respect to the vacuum (Vac) level (i.e., in the absolute energy scale) [39,40,41,42,43].

As described schematically in Figure 1a, AgNPs were synthesized via the PR process on single-layer graphene films (GFs; purity: 97%, Graphene Supermarket, Ronkonkoma, NY, USA) grown by chemical vapor deposition on a Cu surface and transferred onto 285 nm-thick SiO_2_/B-doped Si substrates (resistivity: 0.001–0.005 ohm∙cm) with PMMA (polymethyl methacrylate). Laser illumination was applied to single-layer GFs submerged in 0.1 M AgNO_3_ solution (AgNO_3_ powder purity: >99%, Alfa Aesar, Haverhill, MA, USA). Under the laser illumination, free-electron-hole pairs were generated by laser-assisted photoexcitation, and then Ag ions were reduced to Ag in the form of particles on the graphene surface by accepting the free electrons, releasing oxygen gas molecules [39]. Then, to remove solution residue, the samples were soaked in ultrapure water and then baked at 120 °C for 10 min in air. Figure 1b schematically shows the PR process composed of the charge carrier transport, graphene/solution interfacial charge transfer, and chemical reactions in view of the energy band diagram [40,41,42,43]. The chemical equations for the reactions involved in the PR process are as follows [39]:

(1) $\mathrm{Graphene}\;(G)+h\nu ↔e^{-}\;(G)+h^{+}(G)$,
(2) $e^{-}\;(G)+{\mathrm{AgNO}}_{3}↔\mathrm{Ag}@G+{\mathrm{NO}}_{3}^{-}$,
(3) $h^{+}(G)\;+1/2H_{2}O↔h^{+}(G)+1/2{\mathrm{OH}}^{-}+1/2H^{+}↔1/4O_{2}+H^{+}$, and
(4) $\mathrm{Graphene}\;(G)+h\nu +{\mathrm{AgNO}}_{3}+1/2H_{2}O↔\mathrm{Ag}@G+1/4O_{2}+{\mathrm{HNO}}_{3}$.

**Figure 2:**
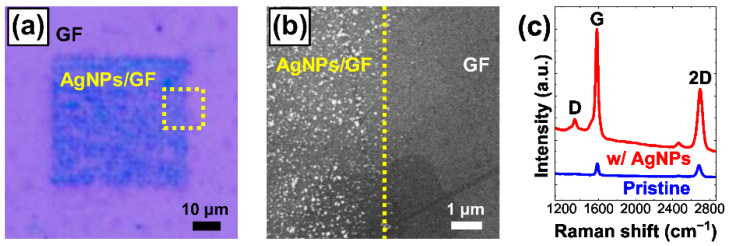
Representative images of AgNPs grown selectively on a graphene film (GF) taken by (**a**) optical microscopy (OM) and (**b**) scanning electron microscop (SEM), respectively. The area marked in the OM image (**a**) was scanned for the SEM image (**b**). (**c**) Raman spectra taken from the same spot of a GF with and without AgNPs [21,33].

Figure 2a shows a representative optical microscopy (OM) image taken from a GF decorated selectively with AgNPs by scanning a focused-laser beam in an area of 50 × 50 μm^2^ on the GF in AgNO_3_ solution at a laser power ($P_{L}$) of 5 mW for a laser dwell time ($\tau_{L}$) of 2000 ms. As shown in Figure 2b, scanning electron microscopy (SEM) imaging showed that the nanoscale particles were distributed only over the laser-processed area. The elemental analysis based on energy-dispersive X-ray spectroscopy (EDS) combined with SEM imaging suggests that the nanoparticles were made of Ag. The details of the EDS analysis are summarized elsewhere (Figure S1).

Figure 2c includes typical Raman spectra acquired from the same spot of a GF before and after the AgNP decoration. The significant enhancement of the overall spectral intensity including 2D and G peaks may be due to the surface-enhanced Raman scattering (SERS) effects, which are well-known to be induced by localized surface plasmon resonances in metal nanoparticles [21,26,29,33,34]. The shift of the peak positions and the changes in the ratios of the peak intensities in Raman spectra represent the variations in microscopic aspects and internal stress states of the graphene as well as the doping effects of the AgNPs into the graphene [13,21,33,44,45,46]. Herein, the peak intensities of the D, G, and 2D bands were set as $I_{D}$, $I_{G}$, and $I_{2D}$ respectively. The increase in the $I_{D}/I_{G}$ intensity ratio by ~81.2% through the PR process indicated the creation of defects in the GF during the AgNP growth [13,21,33,44,45]. In addition, while the 2D and G peaks were blue-shifted by 17.6 cm^−1^ and 1.7 cm^−1^, respectively, the $I_{2D}/I_{G}$ intensity ratio dropped by ~40.6% representing simultaneous doping effects and structural disorder evolution [13,21,33,44,45]. In addition, the blue-shift of the 2D peak may also imply that tensile stress was applied in graphene upon the AgNP decoration [46]. However, no significant change in the Raman characteristics of the graphene was observed after only the focused-laser irradiation in water without AgNP-formation under the laser processing conditions used in this work. This implies that the focused-laser-irradiation itself may have not significantly resulted in microscopic structure change and defect creation in the graphene [13,21,33,44,45,46].

Both the focused-laser irradiation for the PR process and Raman spectroscopy experiments were carried out in the ambient conditions on a confocal Raman spectrometer system (XperRamCompact, NANOBASE, Seoul, Korea) equipped with objective lenses and a continuous-wave laser source of wavelength 532 nm. During the focused-laser irradiation, the laser beam diameter and raster step size were ~5 μm and ~3 μm, respectively, and the $P_{L}$ was set in the range from 2 mW to 30 mW (in the power density ~0.14 MW/cm^2^ to ~2.04 MW/cm^2^). The AgNP growth could be roughly localized down to the spot area of the laser beam (Figure S2). Raman spectra were measured using a laser beam focused to a laser beam diameter of ~1 μm at $P_{L}$ of 1 mW (in the power density ~1.70 MW/cm^2^). The morphology of the AgNPs was observed in detail using SEM equipped with a field-emission gun as an electron source. (JSM-7600F, JEOL, Tokyo, Japan) and analyzed by the ImageJ software. EDS (X-Max, Oxford Instruments, Abingdon, UK) was also conducted for elemental analyses on the surface of AgNPs-decorated graphene using the field-emission electron source of the SEM operated at an accelerating voltage of 15 keV.

### 2.2. Field-Effect-Transistor Device Fabrication and Characterization

GFET devices were built through a series of conventional fabrication processes including photolithography, reactive ion etching with O_2_ plasma, metallization, etc. Top metal electrodes of Cr/Au (thickness: ~5 nm/~50 nm) were deposited onto the patterned graphene films by e-beam evaporation. Then, all the devices were annealed at 350 °C for 15 min while the processing pressure was kept at ~100 mTorr using Ar gas whose flow rate was ~0.5 sccm (standard cubic centimeters per minute) in a rapid thermal annealing system. The GFET devices were back-gated across the 285 nm-thick SiO_2_ layers using the heavily p-doped Si substrates as the bottom electrodes during device characterization with a semiconductor device parameter analyzer (B1500A, Keysight, Santa Rosa, CA, USA) at room temperature and vacuum pressure of ~3 × 10^−6^ Torr in a vacuum probe station equipped with a turbo pump system. To explore the interactive effects of AgNPs on the electronic and electrical properties of graphene as well as the device characteristics of the GFETs, the device measurements were carried out before and after the selective synthesis of AgNPs via the PR process on the microscale graphene channels (GCs).

## 3. Results and Discussion

**Figure 3:**
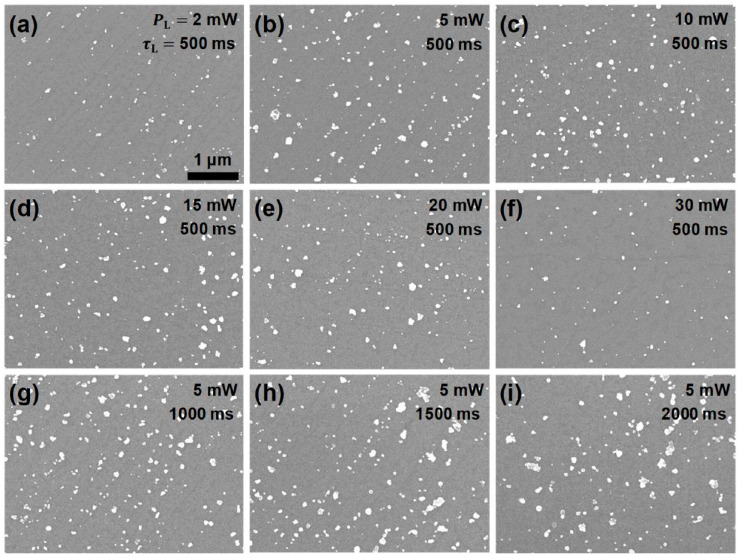
Representative SEM images taken from the regions of AgNPs/GF prepared by the PR process under various focused-laser irradiation conditions: for constant laser dwell time ($\tau_{L}$) of 500 ms, at various laser powers ($P_{L}$s) of (**a**) 2 mW, (**b**) 5 mW, (**c**) 10 mW, (**d**) 15 mW, (**e**) 20 mW, and (**f**) 30 mW, and at a fixed $P_{L}$ of 5 mW for varying $\tau_{L}$s of (**b**) 500 ms, (**g**) 1000 ms, (**h**) 1500 ms, and (**i**) 2000 ms. All images are presented at the same scale.

To investigate how the laser processing parameters affected the growth of the AgNPs, a series of PR experiments were carried out varying the $P_{L}$ and $\tau_{L}$. Then, the AgNPs/GF regions were observed by OM to roughly investigate how the growth of the AgNPs depended on the laser parameters. Selective OM images of the AgNPs/GFs prepared under different laser parameters can be found elsewhere (Figure S3). Then, the morphology and distribution of the AgNPs were probed more precisely through the use of SEM. Figure 3a–f displays a representative set of SEM images of the AgNPs/GF regions given by the PR process operated in the range of $P_{L}$ from 2 mW to 30 mW for a $\tau_{L}$ of 500 ms. In addition, Figure 3b,g–i includes a series of typical SEM images taken from the AgNPs/GF regions prepared at a $P_{L}$ of 5 mW in the range of $\tau_{L}$ from 500 ms to 2000 ms. For statistically reliable analyses of the morphology of the AgNPs, the surface coverage (θ) and average particle size (α) for each laser condition were determined by considering at least five SEM images taken from different AgNPs/GF regions of 3.5 × 5 μm^2^. Herein, α indicates the average diameter of the AgNPs calculated on the assumption that all particles were spherical.

**Figure 4:**
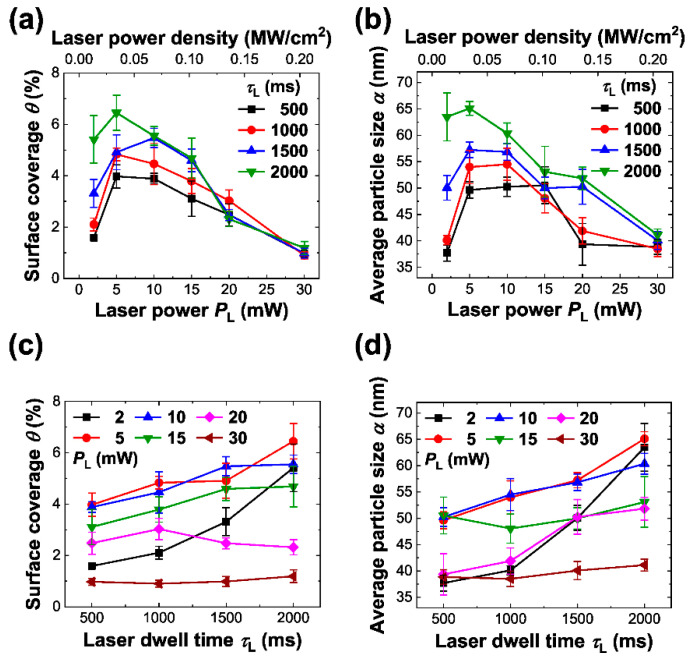
Morphology characteristics of AgNPs grown on a GF at varying laser parameters. (**a**) The surface coverage (θ) and (**b**) average particle size (α) of AgNPs are plotted as a function of $P_{L}$ at various $\tau_{L}s$. (**c**) The θ vs. $\tau_{L}$ and (**d**) α vs. $\tau_{L}$ plots are also given at several different $P_{L}s$. The error bars indicate the standard deviation.

Figure 4a–d exhibits that both of the θ and α of the AgNPs are affected by the $P_{L}$ and $\tau_{L}$ in a complicated way. At the relatively low $P_{L}$ of 5 mW or less, as expected, the AgNPs grew more as the $P_{L}$ or $\tau_{L}$ increased controllably. However, it appeared that excessive laser illumination resulted in the degradation in AgNP growth probably since the reactions were hindered by the convection and evaporation of the solution more heavily with increasing $P_{L}$ or $\tau_{L}$. When the $P_{L}$ was set as 20 mW, the AgNP growth was observed to be the most active at a $\tau_{L}$ of 1000 ms, while at 30 mW, the growth was quite suppressed in the overall range of $\tau_{L}$. Although the laser conditions should have been optimized for growing the AgNPs efficiently, these results imply that, via controlling the laser parameters for the PR process, the growth kinetics of the AgNPs on graphene were delicately adjustable, leading to the detailed tunability of the electronic and electric properties of the AgNP-decorated graphene as well as the device characteristics of the GFETs. Due to the excellent electronic [2] and mechanical properties [3,4] of graphene, the GEET can be embedded into high-performance electronics [47], opto-electronics [26], sensors [48], as well as wearable devices [49].

**Figure 5:**
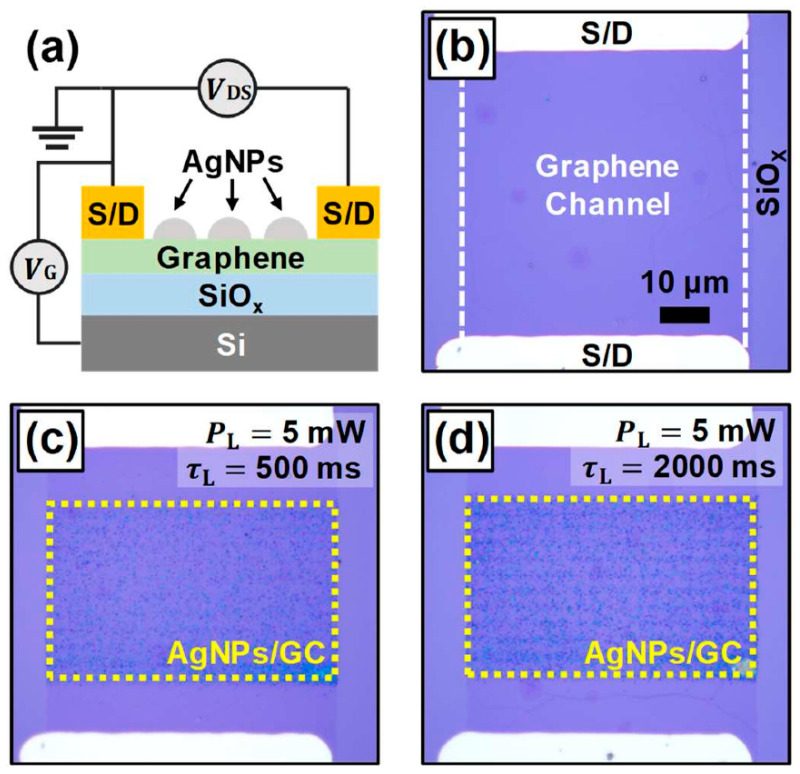
(**a**) Schematic side-view diagram of the graphene-based field effect transistor (GFET) device where the graphene channel (GC) surface is covered with AgNPs. OM plan-view images of the GFET devices with (**b**) an as-fabricated GC and two AgNP-coated GCs prepared differently at $P_{L}$ of 5 mW for $\tau_{L}$s of (**c**) 500 ms (dev1) and (**d**) 2000 ms (dev2), respectively. (**b**–**d**) Are displayed at the same scale.

To investigate how the properties of graphene were affected by the Ag decoration, GFET characterization was carried out at room temperature in conjunction with the PR experiments. Considering that the surface morphology deformation and coalescence of AgNPs are known to occur via surface diffusion actively above 200 °C, the structure of the AgNPs/GC and the GFET performance were expected to be stable under ordinary operational conditions [38,50,51]. The interactions between the GC and AgNPs were investigated by monitoring the device characteristics of the back-gated GFET structure based on a GC illustrated schematically in the side-view in Figure 5a. Figure 5b includes an OM plan-view image showing one of the typical GFET devices exploited in this study where both their channel widths and lengths were ~60 μm. The PR process was applied to the two different GFET devices for selectively decorating the GCs with AgNPs in the area of ~60 × 30 μm^2^ at a $P_{L}$ of 5 mW for a $\tau_{L}$ of 500 ms and 2000 ms, as shown in Figure 5c,d, respectively. Henceforth, the former was called dev1, while the latter was called dev2. The metal electrodes and channel regions near them were not laser-irradiated directly to avoid any damage to the devices. Consistently with the trend observed in Figure 4a–d, it is likely that, at this $P_{L}$ of 5 mW, the longer $\tau_{L}$ led to controllably producing a larger amount of AgNPs in terms of the contrast of the OM images.

**Figure 6:**
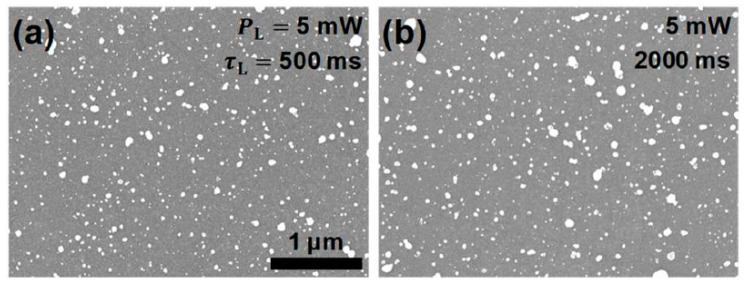
Selective SEM images of the regions of AgNP-decorated GCs. The AgNPs/GC regions in (**a**,**b**) were prepared by the PR process at $P_{L}$ of 5 mW for $\tau_{L}$s of 500 ms (dev1) and 2000 ms (dev2), respectively. Both images are displayed at the same scale.

Further, SEM was also performed on the AgNPs/GCs of dev1 and dev2 to evaluate the morphology of AgNP in more detail and understand their influence on the device characteristics. Figure 6 includes the two representative SEM images scanned from the AgNPs/GC regions of dev1 in Figure 5c and dev2 in Figure 5d. The AgNPs seemed to grow more actively on the patterned GCs than those on the GFs and the reasons are discussed later. Based on the SEM analyses, the θ & α values of the AgNPs on the GCs of dev1 and dev2 were estimated to be ~6.34 ± 0.25% & ~24.72 ± 0.47 nm and ~6.77 ± 0.32% & ~29.60 ± 1.25 nm, respectively. The α values of the AgNPs on the GCs were observed to be generally smaller than those on the GFs under the same laser conditions, whereas the θ values showed the opposite trend (Figure S4). From these results, it can be inferred that initial seeds for AgNP growth may exist more in fabricated GCs than in pristine GFs, as also confirmed by the comparison of the number of AgNPs.

**Figure 7:**
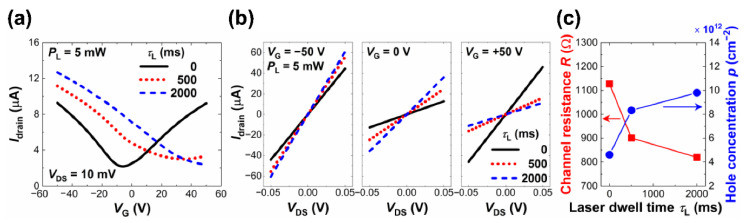
Variations in GFET device characteristics by AgNP decoration. (**a**) Drain current $I_{\mathrm{drain}}$ vs. gate voltage ($V_{G}$) curves measured at $V_{\mathrm{DS}}=$ 10 mV on the GFETs based on GCs decorated without and with AgNPs prepared at $P_{L}$ of 5 mW for varying $\tau_{L}$s. (**b**) Three sets of $I_{\mathrm{drain}}$ vs. $V_{\mathrm{DS}}$ plots of the GFETs with and without AgNP decoration at $V_{G}=$ 0 and ±50 V for each. (**c**) The R and p at $V_{G}=$ −50 V as a function of the $\tau_{L}$.

As shown in Figure 7a, the drain current ($I_{\mathrm{drain}}$) was measured as a function of the gate voltage ($V_{G}$) in the range of −50 V to +50 V at a drain-to-source voltage ($V_{\mathrm{DS}}$) of 10 mV on dev1 before and after the AgNPs were decorated at a $P_{L}$ of 5 mW for a $\tau_{L}$ of 500 ms. For comparison, Figure 7a also includes the $I_{\mathrm{drain}}$ vs. $V_{G}$ plot for dev2 where the AgNPs/GC structure was prepared at the same $P_{L}$ but for a longer $\tau_{L}$ of 2000 ms. Dev1 showed, with the as-fabricated GCs before AgNP decoration, typical ambipolar transport behavior with a small negative Dirac point voltage ($V_{\mathrm{Dirac}}$) indicating that the GC of dev1 was lightly n-doped in the initial stage. It should be noted that dev2 showed very similar $I_{\mathrm{drain}}-V_{G}$ characteristics in the as-fabricated state to those of dev1 (data not shown here).

In addition, the $I_{\mathrm{drain}}$ vs. $V_{\mathrm{DS}}$ curves were acquired in the range of $V_{\mathrm{DS}}$ from −50 mV to +50 mV at various $V_{G}$s for monitoring the $V_{G}$-dependent channel resistance (R) as well as evaluating the performance of the metal-graphene contacts. Figure 7b shows the selective three sets of the $I_{\mathrm{drain}}-V_{\mathrm{DS}}$ data at $V_{G}$ of 0 and ±50 V for each sample. It was clearly observed that the R varied with $V_{G}$ and was influenced by the AgNP decoration, which was consistent with the $I_{\mathrm{drain}}-V_{G}$ behavior shown in Figure 7a. Moreover, the linearity of the $I_{\mathrm{drain}}$ vs. $V_{\mathrm{DS}}$ plots validated the fact that the Ohmic contacts were created with negligible Schottky barrier heights across the metal–graphene interfaces of the GFETs throughout the operation. Considering the correlation between the AgNP morphology characteristics and device behavior, AgNPs formation seemed to enhance the channel current level, particularly in the regime below the $V_{\mathrm{Dirac}}$ where the holes were the dominant charge carriers along with the shift of $V_{\mathrm{Dirac}}$ in the positive direction of $V_{G}$.

These p-type doping effects could be due to the possible transfer of charge carriers such as free electrons from the graphene to the AgNPs (or free holes in the opposite direction) and the resultant lowering of the Fermi energy level in the graphene. Previously, the n-type doping effects in graphene have been reported based on several AgNPs/graphene heterostructures created in different ways from that used in this work [21,26,29,33]. However, considering that the work function of Ag varies in the wide range from 4.14 eV to 4.81 eV due to many factors such as surface crystallographic orientation, crystal structures, synthesis methods, environmental conditions, etc., it is not surprising that p-type doping was achieved here [41,52,53,54,55,56,57]. In addition, the work functions of graphene and SiO_x_ are ~4.60 eV and ~4.90 eV, respectively, and hence, the transfer of electrons from graphene to the SiO_x_/Si substrate possibly contribute to the p-type doping effects [41,56,57]. Rather, the possibility of bidirectional doping with the same nanoparticle material would make this material system attractive for developing more advanced device structures on graphene such as photoactive p-n junctions [13].

As plotted in Figure 7c, the R values in the hole conduction regime at $V_{G}=-50$ V decreased by ~20.1% and ~27.3% via the AgNP decoration grown at a $P_{L}$ of 5 mW for $\tau_{L}$s of 500 ms and 2000 ms, respectively. Figure 7a shows that dev1 experienced a shift of $V_{\mathrm{Dirac}}$ of ~40 V in the positive direction of $V_{G}$ and that of dev2 was even shifted over the upper limit of the $V_{G}$ measurement range ($V_{G}=$ +50 V), confirming that the p-type doping effects became stronger in the graphene as more AgNPs were formed. The field-effect hole mobility ($\mu_{p}$) of the GCs was also calculated as $\mu_{p}=Lg_{m}/WC_{\mathrm{ox}}V_{\mathrm{DS}}$, where W and L are both the width and length of the GCs, respectively, and were ~60 μm, $C_{\mathrm{ox}}$ is the oxide capacitance per area, and $g_{m}$ is the slope of an $I_{\mathrm{drain}}$ vs.$\;V_{G}$ plot in the linear region. The $\mu_{p}$ was observed to decrease with the amount of AgNPs on the GC surface represented by the α value; for dev1, from ~1202.5 cm^2^/V‧s to ~832.9 cm^2^/V‧s (~30.7% decrease), while for dev2, ~1236.1 cm^2^/V‧s to ~777.9 cm^2^/V‧s (~37.1%). The hole carrier concentration (p), defined as the number of holes per unit area, was calculated in the hole conduction regime at $V_{G}=-50$ V, where the holes were the majority of the carriers. Figure 7c shows that the p value was enhanced up to ~104.3% as the $\tau_{L}$ value increased to 2000 ms. Accordingly, it was inferred that the $\mu_{p}$ decreased as the carrier scattering limiting the carrier transport in the GCs became stronger upon the charge transfer from the AgNPs [13].

**Figure 8:**
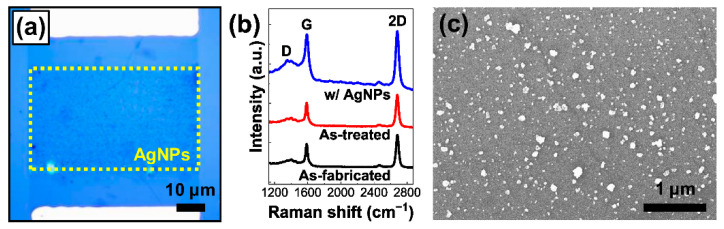
The two-step process composed of laser-irradiation treatment in water and PR-induced AgNP growth. (**a**) OM plan-view image of the GFET device based on a AgNPs/GC prepared by the two-step process (dev3). Both steps were carried out under the same laser conditions of $P_{L}=$ 5 mW and $\tau_{L}=$ 500 ms. (**b**) A set of representative Raman spectra taken from the GC of dev3 in the three states: (i) as-fabricated, (ii) as-treated just after the first step, and (iii) after the second step of AgNP decoration [21,33]. (**c**) A typical SEM image obtained from a AgNPs/GC region of dev3 in (**a**).

Moreover, we also tried a two-step process to prepare a AgNPs/GC where the first step was to apply focused-laser irradiation selectively onto a GC in water, excluding any PR-related chemical reaction, while the second step was, as previously performed, to grow AgNPs on the GC in AgNO_3_ solution by the PR process in the same selective area. The GFET visualized in Figure 8a, called dev3 from now on, underwent the two-step process where both steps were selectively processed at a $P_{L}$ of 5 mW for $\tau_{L}$ of 500 ms in an area of ~60 × 30 μm^2^.

As summarized in Figure 8b, a set of Raman spectra was acquired from the three different GC regions of dev3: (i) as-fabricated, (ii) as-treated just after the first step, and (iii) AgNPs/GC given by the full process. In the comparison of the Raman spectrum taken from the pristine GF in Figure 2c, that of the as-fabricated GC in Figure 8b showed a clear signal of the D peak representing that the graphene seemed to become somehow defective through the device fabrication process [21,33]. The defect sites could act as the seeds for AgNP nucleation, probably leading to more active AgNP growth on the GCs than on the pristine GFs as can be seen above [58]. In addition, no significant change in the Raman characteristics by the first step in Figure 8b may only rule out the possibility of additional severe damage in the graphene by focused-laser irradiation in water and, more importantly, imply that the p-type doping in graphene was mainly due to the interaction with the AgNPs. After the second step, the $I_{D}/I_{G}$ intensity ratio increased by ~71.7% while a decrease of ~ 8.59% in the $I_{2D}/I_{G}$ intensity ratio was observed, indicating additional defect creation and p-type doping effects, respectively [13,21,33,44,45]. Further, an enhancement of the overall Raman spectral intensity was observed from the AgNPs/GC, similarly to the AgNPs/GF in Figure 2c [21,26,29,33,34]. More interestingly, it seems that the two-step process rendered the AgNP growth on the GC more active than the previous one-step process and, accordingly, more substantially enabled the tuning of the device characteristics of the GFETs by AgNP decoration. 

Figure 8c shows a representative SEM image scanned from the AgNPs/GC region of dev3 in Figure 8a. By the SEM image analyses, the θ & α values of the AgNPs/GC of dev3 were given as ~7.43 ± 0.68% & ~32.70 ± 1.46 nm, respectively and they were much higher than those of dev1, i.e., ~6.34 ± 0.25% & ~24.72 ± 0.47 nm, respectively, prepared by the one-step method under the same laser conditions (Figure S4). Probably, the focused-laser treatment in water seemed to enable gentle defect generation as well as surface cleaning and, hence, facilitate more vigorous AgNP growth.

**Figure 9:**
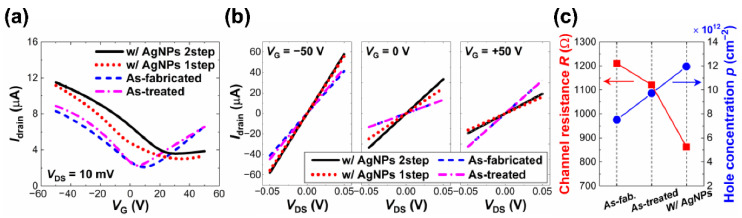
Variations in GFET device characteristics throughout the two-step process. (**a**) Successive measurements of $I_{\mathrm{drain}}$ vs. $V_{G}$ curves of the GFET at $V_{\mathrm{DS}}=$ 10 mV in the three states described previously. (**b**) Sets of $I_{\mathrm{drain}}$ vs. $V_{\mathrm{DS}}$ plots of the GFETs in the three states at $V_{G}=$ 0 and ±50 V for each sample. For the comparison, (**a**,**b**) also include the data of dev1 prepared at a $P_{L}$ value of 5 mW for a $\tau_{L}$ value of 500 ms in Figure 7a,b, respectively, prepared by the one-step process under the same laser conditions. (**c**) The R and p at $V_{G}=-$50 V in the three states.

The tunable characteristics of the GFETs under the two-step process were investigated by measuring the $I_{\mathrm{drain}}-V_{G}$ curve in the range of $V_{G}$ from −50 V to +50 V at a $V_{\mathrm{DS}}$ of 10 mV as well as the $I_{\mathrm{drain}}-V_{\mathrm{DS}}$ data at a $V_{G}$ of 0 and ±50 V from dev3 in the three states of (i) as-fabricated, (ii) as-treated just after the first step, and (iii) AgNPs/GC prepared by the full process, as summarized in Figure 9a,b, respectively. For the comparison, Figure 9a,b also includes the characteristics plots of dev1 prepared by the one-step process at the same $P_{L}$ and $\tau_{L}$. Figure 9c shows that, by the first step, the R of dev3 decreased slightly by ~7.41% at $V_{G}=-50$ V, probably due to the gentle annealing effects driven by the focused-laser irradiation in water. These annealing effects probably enabled the removal of the factors that impair the carrier mobility, such as surface, interface contaminants, adsorbate gas molecules, etc. [59,60]. Then, the decrease in the R of dev3 at $V_{G}=-50$ V was estimated to be ~28.8% throughout the full two-step process, which was much larger than that of the counterpart dev1 (~20.1%) and even that of dev2, which was prepared for a much longer $\tau_{L}$ of 2000 ms (~27.3%). In Figure 9b, the linearity of all the $I_{\mathrm{drain}}$ vs. $V_{\mathrm{DS}}$ plots represents that the quality of the Ohmic contacts were sustained well throughout the full two-step process. The $V_{\mathrm{Dirac}}$ of dev3 was observed to also be shifted in the positive direction of $V_{G}$, similar to dev1 and dev2. Figure 9c shows that p of dev3 reached ~1.19 × 10^13^ cm^−2^ after the two-step process, which was higher than ~8.33 × 10^12^ cm^−2^ of dev1 after the one-step process and even ~9.80 × 10^12^ cm^−2^ of dev2. These results represent that the more active AgNP growth upon the two-step process brought about stronger p-type doping effects on the graphene and, accordingly, more significant modulation of the GFET characteristics.

Moreover, the $\mu_{p}$ values of dev3 were ~687.9 cm^2^/V‧s and ~606.7 cm^2^/V‧s for the as-fabricated and AgNP-decorated GCs, respectively. Although the amount of AgNPs grown on the GC of dev3 was quite a lot larger than that of dev1 in terms of θ, the $\mu_{p}$ of dev3 decreased much less by ~14.1% through the two-step process than that of dev1 (~30.7%). Probably, the additional focused-laser irradiation in water during the first step may have improved the quality of the AgNPs/GC interface and, hence, alleviated the degradation of $\mu_{p}$ by mitigating the influence of interface scattering into the carrier transport, which is particularly evitable in two-dimensional channel-based devices [55,61,62,63]. Based on these results, we expect that it would be possible to further enhance the interaction between graphene and AgNPs by optimizing and engineering the laser-assisted PR process.

## 4. Conclusions

In summary, we demonstrated that a single-layer graphene surface can be decorated area-selectively with AgNPs using the facile one-step focused-laser-assisted PR method and investigated in detail how the formation and growth of AgNPs was affected by the laser-processing parameters. Then, we validated that, based on the GFET device characterization, the selective AgNP decoration on the microscale GFET channel controllably led to p-type doping effects in the graphene. Moreover, we showed that the GFET characteristics could be modulated more substantially with no significant degradation in the carrier mobility in the graphene by carrying out an additional pre-annealing process with a focused-laser beam in advance of the PR process. Therefore, our approach is of relevance to widening the applicability of low-dimensional heterostructures into next-generation electronics and optoelectronics.

## Data Availability

Not applicable.

## Supplementary Materials

The following supporting information can be downloaded at: https://www.mdpi.com/article/10.3390/nano12203549/s1, Figure S1: Energy-dispersive X-ray spectroscopy (EDS) elemental mappings of silver nanoparticles (AgNPs) grown on a graphene film (GF) by the photoreduction (PR) process. (a) EDS spectrum acquired from the AgNPs/GF with the inset including the results of elemental composition analysis. (b) Scanning electron microscopy (SEM) image taken under the EDS mode. The set of elemental maps collected from the area of AgNPs/GF shown in (b) for (c) Si K series, (d) O K series, (e) Ag L series, and (f) C K series. For each elemental map, element-rich and element-deficient regions are presented in bright and dark colors, respectively. The signal of Ag element was detected. Further, only the distribution of Ag element in (e) correlates to some degree with the morphology of NPs shown in (b). Therefore, these results suggest that the AgNPs were formed by the PR process; Figure S2: SEM observation of AgNPs grown locally on a GF by the PR process using focused-laser beam. For the focused-laser irradiation, the laser beam diameter and raster step size were ~5 μm and ~20 μm, respectively, and the laser powers ($P_{L}$) were set at (a,b) 2 mW and (c,d) 5 mW for the two different laser dwell times ($\tau_{L}$s) of 500 ms and 2000 ms. The diameters of AgNP-coated area were determined to be approximately as small as ~5.22 ± 0.38 μm, ~7.53 ± 0.27 μm, ~9.48 ± 0.44 μm, and ~10.03 ± 0.91 μm from the SEM images in (a–d), respectively. In these laser process conditions, AgNPs were observed to be formed in a larger area with a higher $P_{L}$ or a longer $\tau_{L}$. More importantly, it was shown that the growth area of AgNPs could be localized in the level of the laser beam spot size. All SEM images are presented at the same scale; Figure S3: Representative optical microscopy (OM) images of AgNPs selectively grown on a GF in an area of 50 × 50 μm^2^ by the PR process under various laser conditions. The first row of images show the regions of AgNP/GF prepared at the laser powers ($P_{L}$s) of (a) 2 mW, (b) 5 mW, (c) 20 mW, and (d) 30 mW for a laser dwell time ($\tau_{L}$) of 500 ms, while the second row of images display the AgNPs/GF regions given at a fixed $P_{L}$ of 5 mw for varying $\tau_{L}$s of (e) 500 ms, (f) 1000 ms, (g) 1500 ms, and (h) 2000 ms. All images are presented at the same scale; Figure S4: Laser processing parameter-dependent morphology characteristics of AgNP grown on GFs and graphene channels (GCs). (a) The surface coverage (θ) and (b) the average particle size (α) of AgNPs on a GF are plotted as a function of $P_{L}$ at $\tau_{L}$s of 500 ms and 2000 ms. (c) The θ vs. $\tau_{L}$ and (d) α vs. $\tau_{L}$ plots are also given at $P_{L}$s of 2 mW and 5 mW. For the comparison with the θ and α of AgNPs on GCs, the data estimated from the devices (dev1-3) considered in this work are also indicated together. The error bars in (a–d) indicate the standard deviation.
