# Supplementary material for: Tailorable Electronic and Electric Properties of Graphene with Selective Decoration of Silver Nanoparticles by Laser-Assisted Photoreduction
Source: Nanomaterials (Basel). 2022 Oct 11;12(20):3549. doi: 10.3390/nano12203549 (PMC9608314; doi:10.3390/nano12203549)
Supplement: Supplementary file 1 [file nanomaterials-12-03549-s001.zip › nanomaterials-1953665-supplementary.pdf]

nanomaterials

Supplementary Material

Tailorable Electronic and Electric Properties of Graphene with Selective Decoration of Silver Nanoparticles by Laser-Assisted Photoreduction

Inseon Song [1], Yujeong Kim [1], Byung Hoon Lee [2], Minji Chae [1], Sooyeon Kim [1], ChangKyu Yoon [3,4], Min-Kyu Joo [1,4], Jeeyoung Shin [3,4], Soo Min Kim [5,*] and Changhyun Ko [1,4,*]

[1] Department of Applied Physics, College of Engineering, Sookmyung Women's University, Seoul 04310, Korea
[2] Center for Integrated Nanostructure Physics (CINAP), Institute for Basic Science (IBS), Sungkyunkwan University, Suwon 16419, Korea
[3] Department of Mechanical Systems Engineering, Sookmyung Women's University, Seoul 04310, Korea
[4] Institute of Advanced Materials and Systems, Sookmyung Women's University, Seoul 04310, Korea
[5] Department of Chemistry, Sookmyung Women's University, Seoul 04310, Korea
* Correspondence: soominkim@sookmyung.ac.kr (S.M.K.); cko@sookmyung.ac.kr (C.K.)

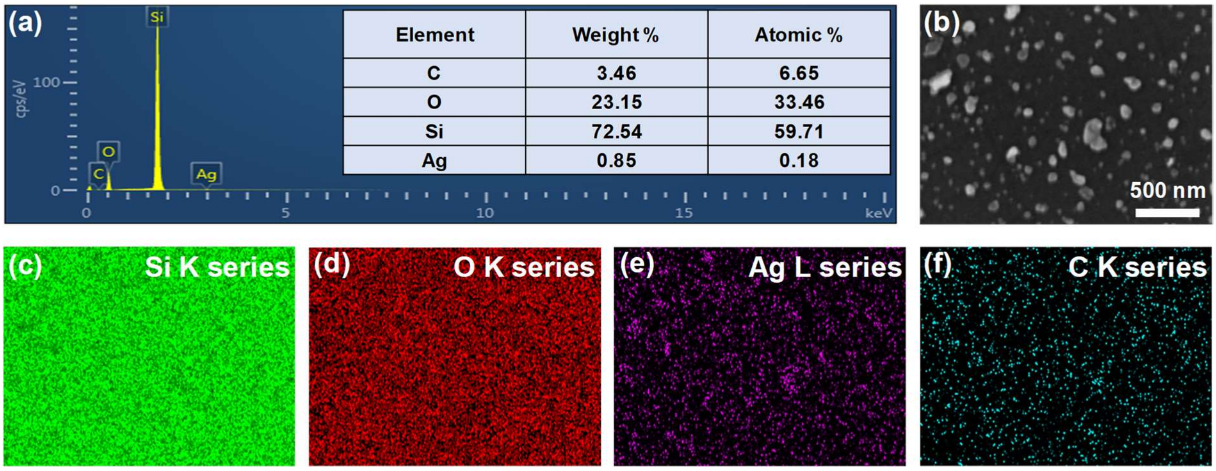

Figure S1. Energy-dispersive X-ray spectroscopy (EDS) elemental mappings of silver nanoparticles (AgNPs) grown on a graphene film (GF) by the photoreduction (PR) process. **(a)** EDS spectrum acquired from the AgNPs/GF with the inset including the results of elemental composition analysis. **(b)** Scanning electron microscopy (SEM) image taken under the EDS mode. The set of elemental maps collected from the area of AgNPs/GF shown in (b) for **(c)** Si K series, **(d)** O K series, **(e)** Ag L series, and **(f)** C K series. For each elemental map, element-rich and element-deficient regions are presented in bright and dark colors, respectively. The signal of Ag elements was detected. Further, only the distribution of Ag element in (e) correlates to some degree with the morphology of NPs shown in (b). Therefore, these results suggest that the AgNPs were formed by the PR process.

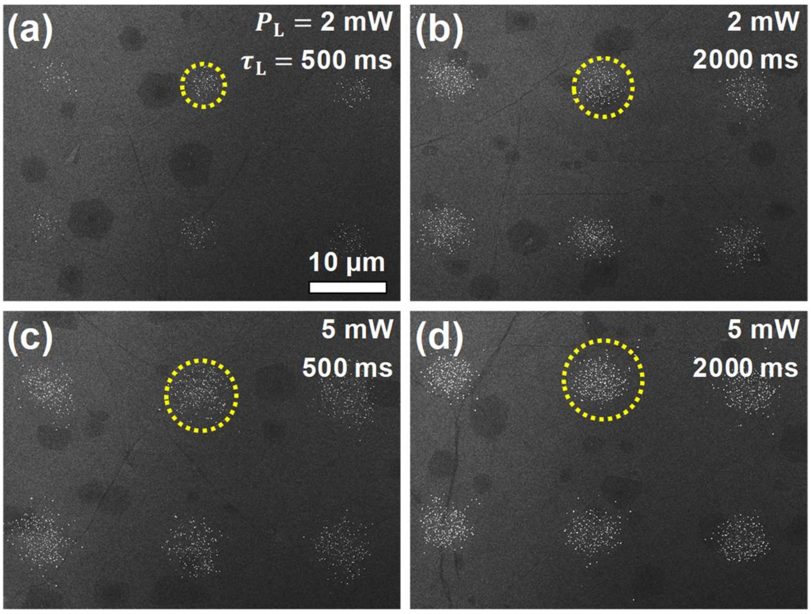

Figure S2. SEM observation of AgNPs grown locally on a GF by the PR process using focused-laser beam. For the focused-laser irradiation, the laser beam diameter and raster step size were ~ 5 μm and ~ 20 μm, respectively, and the laser powers (P_L) were set at **(a)-(b)** 2 mW and **(c)-(d)** 5 mW for the two different laser dwell times (τ_Ls) of 500 ms and 2000 ms. The diameters of AgNP-coated area were determined to be approximately as small as ~ 5.22 ± 0.38 μm, ~ 7.53 ± 0.27 μm, ~ 9.48 ± 0.44 μm, and ~ 10.03 ± 0.91 μm from the SEM images in (a)-(d), respectively. In these laser process conditions, AgNPs were observed to be formed in a larger area with a higher P_L or a longer τ_L. More importantly, it was shown that the growth area of AgNPs could be localized in the level of the laser beam spot size. All SEM images are presented at the same scale.

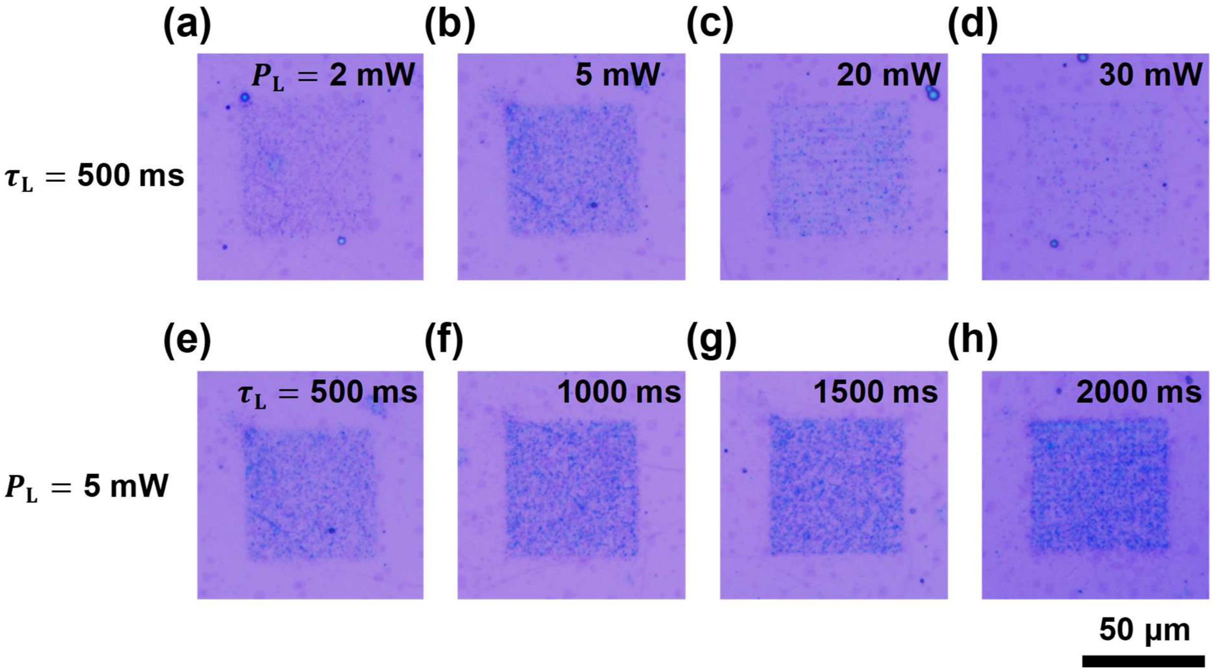

Figure S3. Representative optical microscopy (OM) images of AgNPs selectively grown on a GF in an area of 50 × 50 μm^2 by the PR process under various laser conditions. The first row of images show the regions of AgNP/GF prepared at the laser powers (P_Ls) of **(a)** 2 mW, **(b)** 5 mW, **(c)** 20 mW, and **(d)** 30 mW for a laser dwell time (τ_L) of 500 ms, while the second row of images display the AgNPs/GF regions given at a fixed P_L of 5 mw for varying τ_Ls of **(e)** 500 ms, **(f)** 1000 ms, **(g)** 1500 ms, and **(h)** 2000 ms. All images are presented at the same scale.

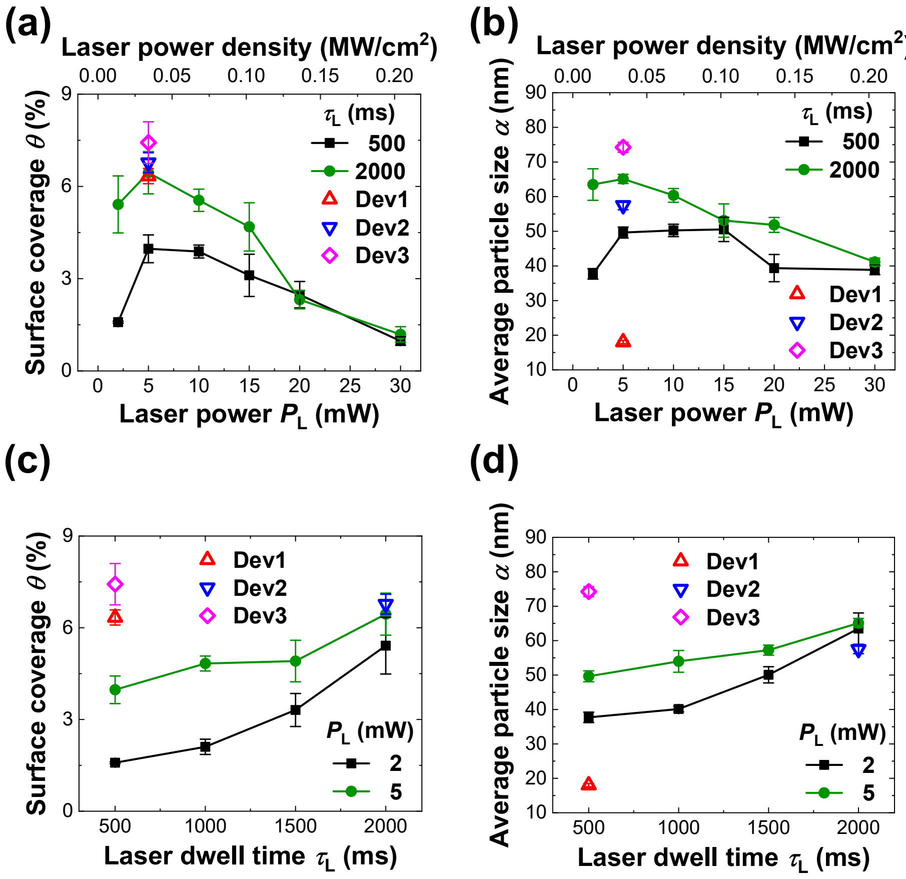

Figure S4. Laser processing parameter-dependent morphology characteristics of AgNP grown on GFs and graphene channels (GCs). **(a)** The surface coverage (θ) and **(b)** the average particle size (α) of AgNPs on a GF are plotted as a function of P_L at τ_Ls of 500 ms and 2000 ms. **(c)** The θ vs. τ_L and **(d)** α vs. τ_L plots are also given at P_Ls of 2 mW and 5 mW. For the comparison with the θ and α of AgNPs on GCs, the data estimated from the devices (dev1-3) considered in this work are also indicated together. The error bars in (a)-(d) indicate the standard deviation.
